# Phase Transitions and Thermoelectric Properties of Charge-Compensated Zn_x_Cu_12−x_Sb_4_Se_13_

**DOI:** 10.3390/ma17133282

**Published:** 2024-07-03

**Authors:** Sang Jun Park, Il-Ho Kim

**Affiliations:** Department of Materials Science and Engineering, College of Engineering, Korea National University of Transportation, Chungju 27469, Republic of Korea; psj8479@naver.com

**Keywords:** thermoelectric, hakite, charge compensation

## Abstract

In this study, we investigated the phase transitions and thermoelectric properties of charge-compensated hakite (Zn_x_Cu_12−x_Sb_4_Se_13_) as a function of Zn content. Based on X-ray diffraction and a differential scanning calorimetric phase analysis, secondary phases (permingeatite and bytizite) transformed into hakite depending on the Zn content, while Zn_2_Cu_10_Sb_4_Se_13_ existed solely as hakite. Nondegenerate semiconductor behavior was observed, exhibiting increasing electrical conductivity with a rising temperature. With an increase in Zn content, the presence of mixed phases of hakite and permingeatite led to enhanced electrical conductivity. However, Zn_2_Cu_10_Sb_4_Se_13_ with a single hakite phase exhibited the lowest electrical conductivity. The Seebeck coefficient exhibited positive values, indicating that even after charge compensation (electron supply) by Zn, p-type semiconductor characteristics were maintained. With the occurrence of an intrinsic transition within the measured temperature range, the Seebeck coefficient decreased as the temperature increased; at a certain temperature, Zn_2_Cu_10_Sb_4_Se_13_ exhibited the highest value. Thermal conductivity showed a low temperature dependence, obtaining low values below 0.65 Wm^−1^K^−1^. A power factor of 0.22 mWm^−1^K^−2^ and dimensionless figure of merit of 0.31 were achieved at 623 K for ZnCu_11_Sb_4_Se_13_.

## 1. Introduction

Thermoelectric devices are semiconductor systems capable of directly converting electrical energy into thermal energy for cooling/heating or recovering waste heat into electricity for power generation; thus, they have gained attention for the provision of green energy and contributing to the reduction of CO_2_ gas [1]. Enhancing the performance of thermoelectric devices is crucial and achieved by maximizing a dimensionless figure of merit (ZT = α^2^σκ^−1^T) with optimized material parameters (α: Seebeck coefficient, σ: electrical conductivity, and κ: thermal conductivity) at the application temperature (T in Kelvin) [2]. α^2^σ is referred to the power factor (PF), and a higher ZT indicates higher energy conversion efficiency. This means that good thermoelectric materials have high electrical conductivity and a Seebeck coefficient, as well as low thermal conductivity [3,4].

Methods to enhance ZT include attempts to optimize specific parameters and the introduction of nanostructures [5]. Another approach is to search for new materials that intrinsically have low thermal conductivity due to the anharmonicity of lattice vibrations (phonons), possessing a regularly ordered crystalline structure [6,7,8,9,10,11,12]. Compounds such as Cu_5_FeS_4_, Cu_3_SbS_4_, and Cu_2_SnSe_3_, which are ternary compounds of Cu–M–Q (M = Sb, Sn, and Fe, and Q = S and Se), are gaining attention for their environmentally friendly and economical nature, and inherently low thermal conductivity. A prominent example for a novel thermoelectric material is the tetrahedrite Cu_12_Sb_4_S_13_, which exhibits a narrow bandgap, a cubic structure, and is abundant in the earth [13,14,15,16,17].

Johan and Kvaček [18] firstly named the analogue of tetrahedrite Cu_12_Sb_4_S_13_, where Se substitutes for S, as hakite Cu_12_Sb_4_Se_13_. Biagioni et al. [19] considered the valences, occupancies, and types of elements to satisfy charge neutrality, determining the stability of hakite as ${Cu}_{6}^{+}[{Cu}_{4}^{+}{Cu}_{2}^{2+}]{Sb}_{4}^{3+}{Se}_{12}^{2-}{Se}^{2-}$. Škácha et al. [20,21] described the presence of hakite, highlighting its prevalence with Hg^2+^, Cd^2+^, Zn^2+^, Fe^2+^, and Cu^2+^. Karup-Møller and Makovicky [22] claimed the ability to create charge-compensated Fe-hakite, Cu_6_[Cu_4_Fe_2_]Sb_4_Se_13_ (Fe_2_Cu_10_Sb_4_Se_13_) and Zn-hakite, Cu_6_[Cu_4_Zn_2_]Sb_4_Se_13_ (Zn_2_Cu_10_Sb_4_Se_13_). However, they reported that synthesis of cubic hakite, Cu_6_[Cu_4_Cu_2_]Sb_4_Se_13_ (Cu_12_Sb_4_Se_13_), was not possible and, instead, orthorhombic bytizite (Cu_3_SbSe_3_) was formed. In this study, we attempted the synthesis of Zn-compensated hakite (Zn_x_Cu_12−x_Sb_4_Se_13_) and investigated the phase transitions and thermoelectric properties as a function of Zn content. Our goal was to provide experimental data on the hakite.

## 2. Experimental Procedure

Zn-hakite, Zn_x_Cu_12−x_Sb_4_Se_13_ (x = 0.5, 1, 1.5, and 2), where Zn^2+^ compensates for Cu^2+^ (and actually exists as Cu^+^), was synthesized. Mechanical alloying (MA) was employed to homogeneously synthesize compounds and prevent volatilization. Zn (purity 99.9%, <75 μm), Cu (purity 99.9%, <45 μm), Sb (purity 99.999%, <150 μm), and Se (purity 99.9%, <10 μm) elemental powders were used for MA. A planetary ball mill (Pulverisette5, Fritsch, Pittsboro, NC, USA) consisting of stainless steel balls and vessels was utilized. The interior of the vessel was evacuated and then filled with Ar gas, and MA was conducted at 350 rpm for 24 h. The synthesized powder was packed into graphite molds and subjected to hot pressing (HP) under vacuum conditions at 623 K for 2 h with a pressure of 70 MPa. The optimized MA–HP process conditions were determined in our preliminary study [23].

X-ray diffraction (XRD; D8-Advance, Bruker, Billerica, MA, USA) was employed to analyze the phases of the MA powders and HP-sintered bodies using Cu Kα radiation. A thermogravimetric analysis and differential scanning calorimetry (TG–DSC; TGA/DSC1, Mettler Toledo, Columbus, OH, USA) were utilized to investigate the phase transitions, specific heat, and thermal stability of Zn-hakite. For microstructural observation, scanning electron microscopy (SEM; Quanta400, FEI, Lausanne, Switzerland) in backscattered electron (BSE) mode was utilized. Energy-dispersive X-ray spectroscopy (EDS; Quantax200, Bruker) was used for elemental distribution and the compositional analysis. The ZEM-3 (Advance Riko, Yokohama, Japan) instrument was used to measure the Seebeck coefficient and electrical conductivity using the DC four-probe method under He atmosphere. TC-9000H (Advance Riko) equipment was employed to determine thermal diffusivity (D) using the laser flash method in vacuum, and thermal conductivity (κ = dc_p_D) was evaluated using measured density (d) and specific heat (c_p_). Measurements were conducted in the temperature range of 323–623 K, and the PF and ZT were evaluated based on the electrical conductivity, Seebeck coefficient, and thermal conductivity.

## 3. Results and Discussion

**Figure 1:**
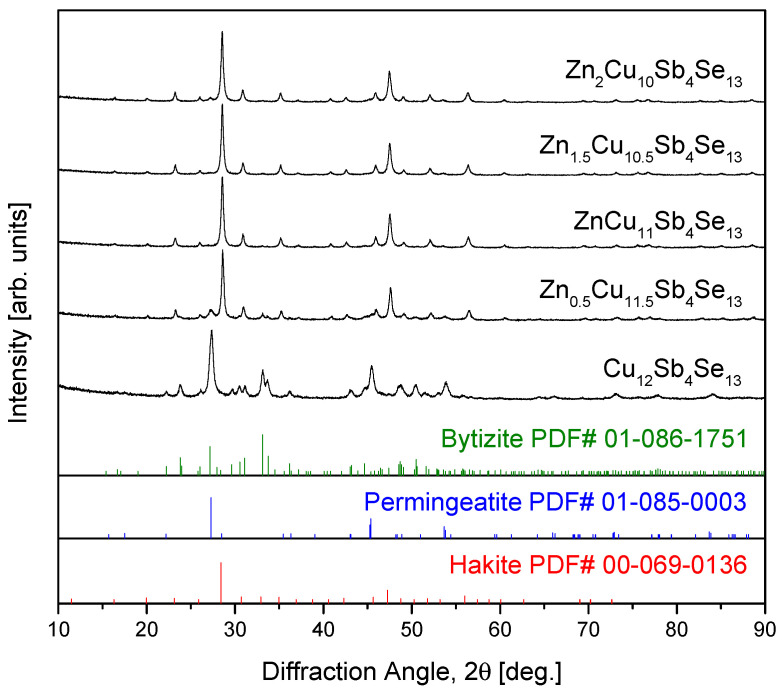
XRD patterns of Zn_x_Cu_12−x_Sb_4_Se_13_ synthesized by MA.

Figure 1 shows the XRD phase analysis results of the MA-synthesized Zn_x_Cu_12−x_Sb_4_Se_13_ powders. For the sample uncompensated with Zn (x = 0), the hakite phase was not produced due to electronic instability, but permingeatite (PDF# 01-085-0003; tetragonal Cu_3_SbSe_4_; a = 0.56609 nm and c = 1.12800 nm) and bytizite (PDF# 01-086-1751; orthorhombic Cu_3_SbSe_3_; a = 0.79865 nm, b = 1.06138 nm, and c = 0.68372 nm) were formed [23]. For the specimen with the Zn content of x = 0.5, hakite (PDF# 00-069-0136; cubic Cu_12_Sb_4_Se_13_; a = 1.08783 nm) was predominantly formed with some presence of permingeatite, but bytizite was not detected. The lattice constants of the phases present in the MA powders were determined as follows: a = 0.54008 nm and c = 1.08567 nm for permingeatite; a = 0.81409 nm, b = 1.14658 nm, and c = 0.73873 nm for bytizite; and a = 1.08149 nm for hakite. The Lorentzian crystallite sizes were calculated to be 31.5 nm for permingeatite, 15.8 nm for bytizite, and 30.2 nm for hakite. With a further increase in Zn content, the secondary phase of permingeatite diminishes, transforming into a single phase of hakite. This stabilization of the hakite phase was attributed to charge compensation through the introduction of Zn^2+^ [20]. As mentioned in the Introduction, Karup-Møller and Makovicky [22] also failed to synthesize pure hakite and instead generated bytizite. Therefore, it reconfirmed that uncompensated pure hakite Cu_12_Sb_4_Se_13_ cannot exist. O et al. [24] discovered different phase transitions in MA-synthesized Fe_x_Cu_12−x_Sb_4_Se_13_; when the Fe content was 0.5 ≤ x ≤ 1, bytizite and eskebornite (PDF# 01-081-1959; tetragonal CuFeSe_2_; a = 0.55210 nm and c = 1.10420 nm) were formed, whereas for 1.5 ≤ x ≤ 2, pribramite (PDF# 01-083-9473; orthorhombic CuSbSe_2_; a = 0.62988 nm, b = 0.39810 nm, and c = 1.50030 nm) was additionally generated. To our best knowledge, apart from Zn-hakite, we have not discovered the formation of any other synthetic hakite.

**Figure 2:**
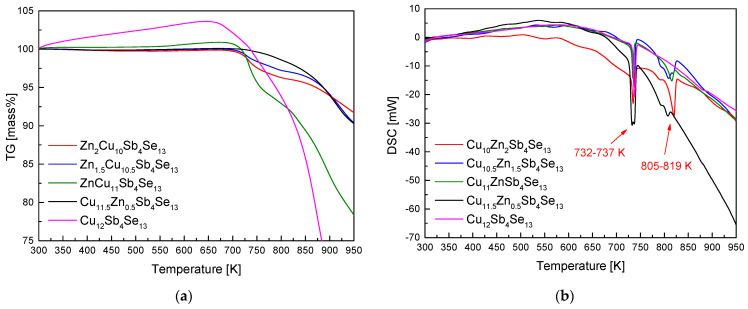
Analyses of (**a**) TG and (**b**) DSC for Zn_x_Cu_12−x_Sb_4_Se_13_ synthesized by MA.

The TG–DSC analysis results for the Zn_x_Cu_12−x_Sb_4_Se_13_ powder are shown in Figure 2. Figure 2a reveals a pronounced mass loss at approximately 723 K, attributed to the volatilization of its constituent elements (notably Se) as a result of phase decomposition and melting. Figure 2b displays two endothermic peaks within the ranges of 732–737 K and 805–819 K, which are interpreted as the melting points of permingeatite and hakite, respectively. The enthalpies of the endothermic reactions of the phases present in the MA powders were measured to be 31.3–59.3 Jg^−1^ for permingeatite and 16.2–54.7 Jg^−1^ for hakite. The DSC results exhibited little differences with varying Zn content. Zhang et al. [25] predicted 18 types of theoretically possible Cu–Sb–Se phases using density-functional theory (DFT); while the melting point of Cu_3_SbSe_4_ is around 700 K [26], it becomes unstable and disappears at 910 K, transforming into a hypothetical Cu_12_Sb_4_Se_13_ phase at 1100 K.

**Figure 3:**
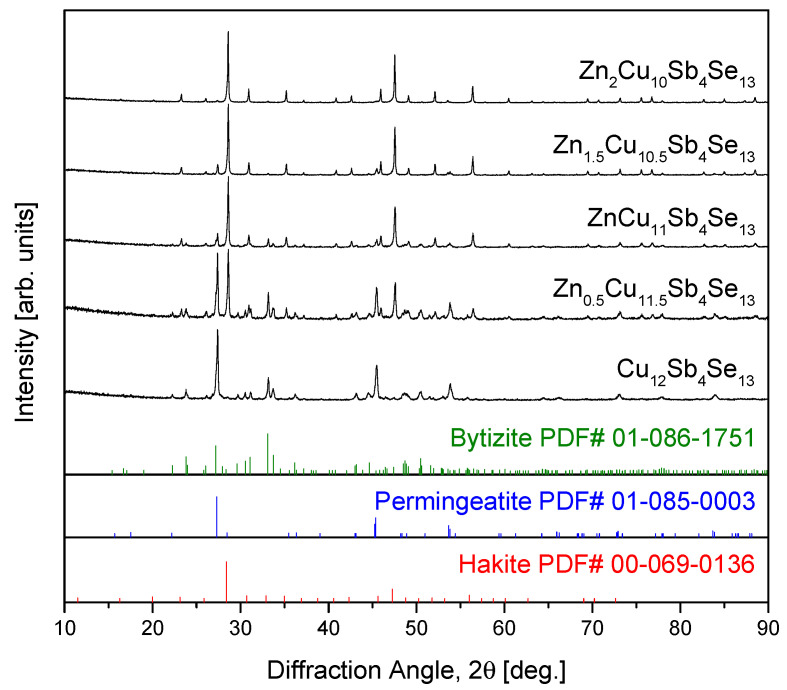
XRD patterns of Zn_x_Cu_12−x_Sb_4_Se_13_ prepared by MA–HP.

Figure 3 depicts the XRD analysis results of Zn_x_Cu_12−x_Sb_4_Se_13_ prepared via MA–HP. Similar to the phases for the MA powders in Figure 1, the diffraction peaks for each phase were sharpened. This was attributed to stress relief and grain growth of the MA powders during the HP process. The lattice constants of the phases present in the HP samples were determined as follows: for permingeatite, a = 0.54089 nm and c = 1.08285 nm; for bytizite, a = 0.81650 nm, b = 1.15100 nm, and c = 0.73972 nm; and for hakite, a = 1.08265 nm. The Lorentzian crystallite sizes were calculated to be 142.0 nm for permingeatite, 16.8 nm for bytizite, and 63.2 nm for hakite. As the Zn content increased, the intensities of diffraction peaks corresponding to permingeatite and bytizite decreased or disappeared, confirming transformation into stable hakite. O et al. [24] reported their XRD analysis of Fe_x_Cu_12−x_Sb_4_Se_13_ prepared via MA–HP, finding that it existed as a composite of bytizite–eskebornite–pribramite phases. They observed a decrease in the amount of bytizite and an increase in pribramite as the Fe content increased, and concluded that Fe-hakite is unstable.

**Figure 4:**
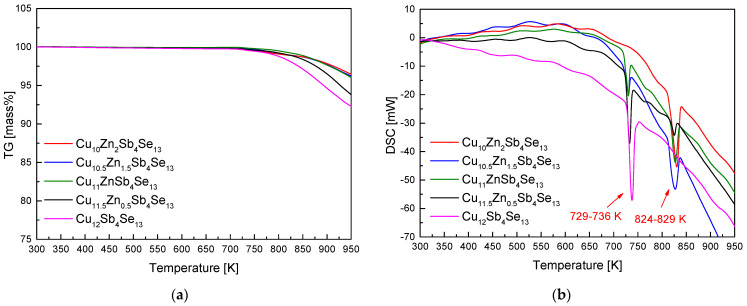
Analyses of (**a**) TG and (**b**) DSC for Zn_x_Cu_12−x_Sb_4_Se_13_ prepared by MA–HP.

Figure 4 shows the TG–DSC curves of Zn_x_Cu_12−x_Sb_4_Se_13_ prepared via MA–HP. A mass loss at temperatures above approximately 723 K was observed in Figure 4a, but the mass loss at high temperatures was significantly lower compared to the MA powder samples in Figure 2a. This was attributed to the reduction in the specific surface area of the sintered bodies and increased phase stability. Two endothermic reactions at 729–736 K and 824–829 K are shown in Figure 4b, consistent with the melting points of permingeatite and hakite as interpreted from Figure 2b. The enthalpies of the endothermic reactions of the phases present in the HP samples were measured to be 11.8–34.5 Jg^−1^ for permingeatite and 8.0–67.6 Jg^−1^ for hakite. The melting point of Zn-hakite showed a slight increase due to HP, attributed to phase stabilization and heat-treatment effects (stress reduction). As Zn content increased, the endothermic peak corresponding to permingeatite diminished, indicating a transformation into hakite. O et al. [24] observed three endothermic peaks at 674–676 K, 712 K, and 720–736 K in their DSC analysis of Fe_x_Cu_12−x_Sb_4_Se_13_ prepared via MA–HP. They interpreted these peaks as melting points corresponding to bytizite, pribramite, and permingeatite, respectively.

**Figure 5:**
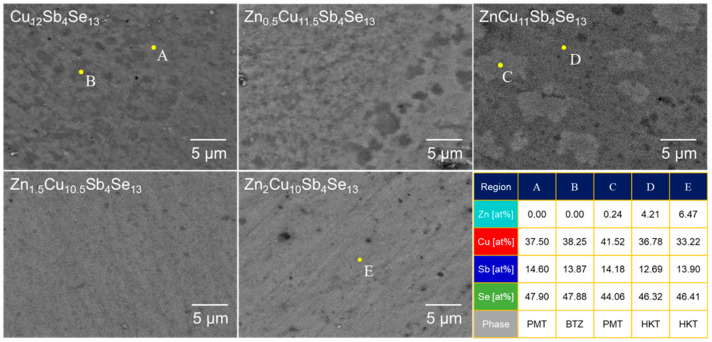
BSE–SEM micrographs and EDS spot analysis of Zn_x_Cu_12−x_Sb_4_Se_13_.

Figure 5 presents the BSE–SEM microstructures and EDS spot analysis for Zn_x_Cu_12−x_Sb_4_Se_13_. In the compositional table, PMT, BTZ, and HKT represent permingeatite, bytizite, and hakite phases, respectively. For Cu_12_Sb_4_Se_13_, permingeatite (region A: bright area) and bytizite (region B: dark area) were observed, with hakite not being formed. Substituting Zn partially for Cu allowed for the presence of the hakite phase in the case of ZnCu_11_Sb_4_Se_13_; the bright area (region C) was identified as permingeatite and the dark area (region D) as hakite. Zn_2_Cu_10_Sb_4_Se_13_ contained a single phase of hakite. This aligned with the results of the XRD phase analysis and DSC thermal analysis.

**Figure 6:**
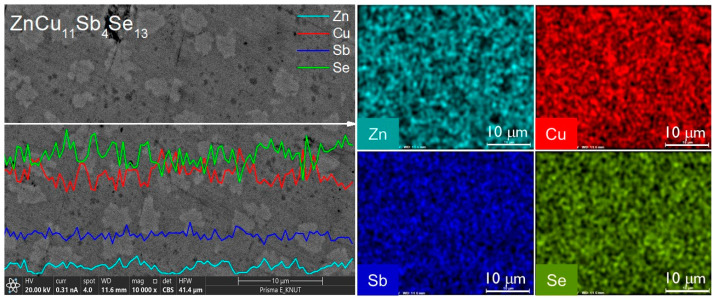
EDS spectra and maps of ZnCu_11_Sb_4_Se_13_.

Figure 6 displays the results of the EDS analysis for ZnCu_11_Sb_4_Se_13_. Line scans revealed the bright area as permingeatite, where a decrease in Zn content and an increase in Cu content were noted, leading to the determination of this area as permingeatite. This was further supported by elemental mapping, where the near absence of Zn content in the bright area reinforced this conclusion.

**Figure 7:**
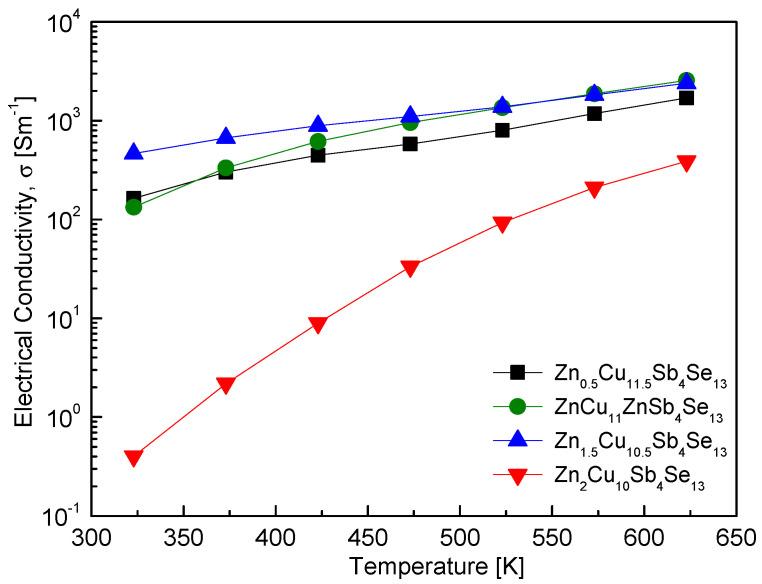
Temperature dependence of the electrical conductivity for Zn_x_Cu_12−x_Sb_4_Se_13_.

The thermoelectric properties of samples prepared via MA–HP were measured, but those of Cu_12_Sb_4_Se_13_ (without the hakite phase) were not investigated. Figure 7 shows the electrical conductivity of Zn_x_Cu_12−x_Sb_4_Se_13_. Positive temperature dependence indicated the behavior of a nondegenerate semiconductor. In samples containing permingeatite (x = 0.5–1.5), in other words, hakite–permingeatite composites, the electrical conductivity increased from 133–465 Sm^−1^ at 323 K to 1698–2561 Sm^−1^ at 623 K. However, in the case of Zn_2_Cu_10_Sb_4_Se_13_ with a single phase of hakite, the electrical conductivity significantly increased from 0.49 Sm^−1^ at 323 K to 387 Sm^−1^ at 623 K. Nevertheless, it exhibited the lowest electrical conductivity at all measured temperatures, attributed to the substitution of Cu^+^ by Zn^2+^, which supplied electrons (hakite being a p-type semiconductor), leading to charge compensation and a decrease in the majority-carrier concentration. Zhang et al. [26] applied the DFT to simulate the band structure of hakite, revealing that the presence of Zn in Cu_12_Sb_4_Se_13_ shifted the Fermi level within the bandgap (reducing hole concentration), and predicted a bandgap of 0.66 eV for Zn_2_Cu_10_Sb_4_Se_13_. In our previous study [23], we confirmed that Zn-hakite (Zn_2_Cu_10_Sb_4_Se_13_) is a p-type semiconductor and reported a carrier concentration of 4.79 × 10^18^ cm^−3^. O et al. [24] found that the electrical conductivity of Fe_x_Cu_12−x_Sb_4_Se_13_ did not vary significantly with Fe content, but they discovered a temperature dependence of a degenerate semiconductor; Fe_2_Cu_10_Sb_4_Se_13_ exhibited the highest electrical conductivity of 583.3 Sm^−1^ at 623 K.

**Figure 8:**
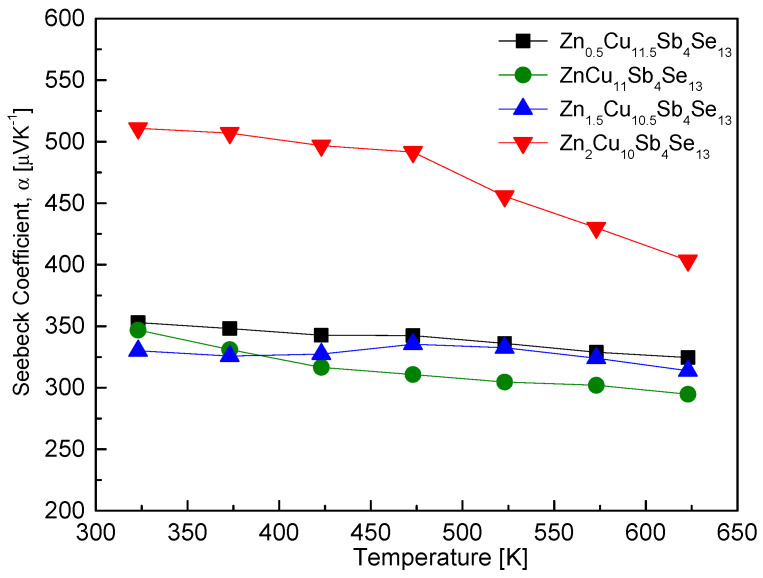
Temperature dependence of the Seebeck coefficient for Zn_x_Cu_12−x_Sb_4_Se_13_.

Figure 8 shows the Seebeck coefficient of Zn_x_Cu_12−x_Sb_4_Se_13_. The positive values of the Seebeck coefficient at all temperature ranges reconfirmed p-type semiconductor characteristics. As the temperature increased, the Seebeck coefficient decreased due to the occurrence of intrinsic transition. Samples with x = 0.5–1.5 showed relatively low temperature-dependencies, with values ranging from 330–353 μVK^−1^ at 323 K to 295–324 μVK^−1^ at 623 K. However, for Zn_2_Cu_10_Sb_4_Se_13_, a significantly high Seebeck coefficient of 511 μVK^−1^ was observed at 323 K, with temperature dependency decreasing to 403 μVK^−1^ at 623 K. O et al. [24] reported a negative temperature dependence of the Seebeck coefficient in Fe_x_Cu_12−x_Sb_4_Se_13_, noting that the variation of the Seebeck coefficient with Fe content was small. They observed a decrease from 641–761 μVK^−1^ at 323 K to 387–401 μVK^−1^ at 623 K.

**Figure 9:**
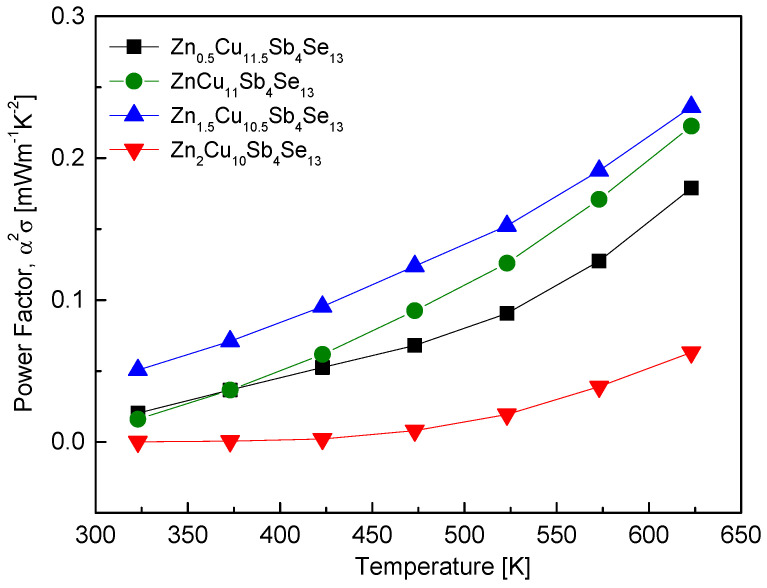
Temperature dependence of the power factor for Zn_x_Cu_12−x_Sb_4_Se_13_.

Figure 9 displays the PF values of Zn_x_Cu_12−x_Sb_4_Se_13_. As the temperature increased, the PF sharply rose, resulting from a significant temperature dependence of electrical conductivity compared to the Seebeck coefficient. With increasing Zn content, the PF also increased, reaching a maximum of 0.24 mWm^−1^K^−2^ at 623 K for Zn_1.5_Cu_10.5_Sb_4_Se_13_. However, for Zn_2_Cu_10_Sb_4_Se_13_, the PF significantly decreased to 0.056 mWm^−1^K^−2^ at 623 K. This was attributed to the necessity of charge compensation with Zn for hakite phase stabilization, while leading to a drastic reduction in electrical conductivity due to the decrease in carrier concentration, resulting in a decrease in the PF. Therefore, the optimal Zn content for improving the PF was determined to be 1 ≤ x ≤ 1.5. O et al. [24] reported that Fe_1.5_Cu_10.5_Sb_4_Se_13_ exhibited a maximum PF of 0.08 mWm^−1^K^−2^ at 623 K in Fe_x_Cu_12−x_Sb_4_Se_13_. They attributed this to the increased effect of electrical conductivity outweighing the decrease in the Seebeck coefficient with increasing temperature.

**Figure 10:**
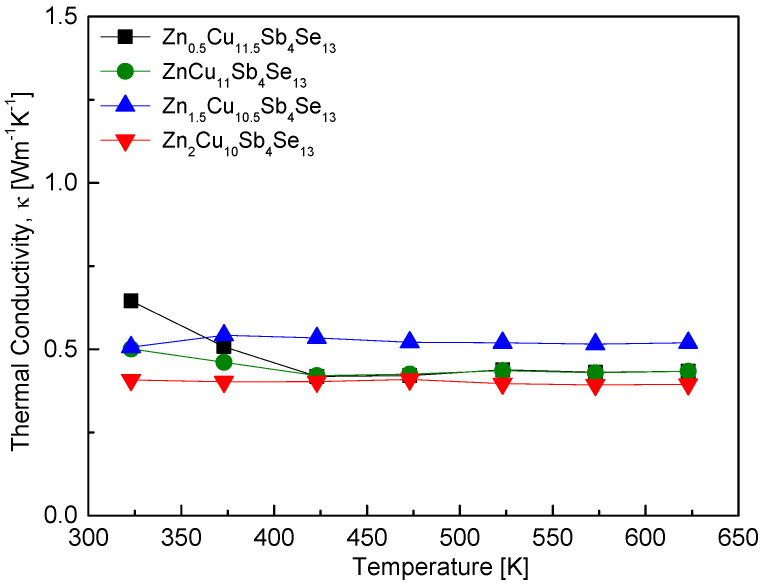
Temperature dependence of the thermal conductivity for Zn_x_Cu_12−x_Sb_4_Se_13_.

Figure 10 presents the thermal conductivity of Zn_x_Cu_12−x_Sb_4_Se_13_. Very low thermal conductivity values were obtained over the temperature range of 323–623 K, with little temperature dependence, ranging from 0.44–0.65 Wm^−1^K^−1^ at 323 K to 0.41–0.52 Wm^−1^K^−1^ at 623 K. The specific heat capacity (c_p_ = 0.3435 Jg^−1^K^−1^) used for thermal conductivity calculations was obtained from our previous study [23]. The theoretical value of pure hakite Cu_12_Sb_4_Se_13_ calculated using the Dulong–Petit law is c_p_ = 0.33 Jg^−1^K^−1^. O et al. [24] reported that the thermal conductivity values of Fe_x_Cu_12−x_Sb_4_Se_13_ were 0.42–0.79 Wm^−1^K^−1^ at 323 K and 0.33–0.54 Wm^−1^K^−1^ at 623 K, and found that Fe_0.5_Cu_11.5_Sb_4_Se_13_ exhibited a minimum thermal conductivity of 0.30 Wm^−1^K^−1^ at 423 K.

**Figure 11:**
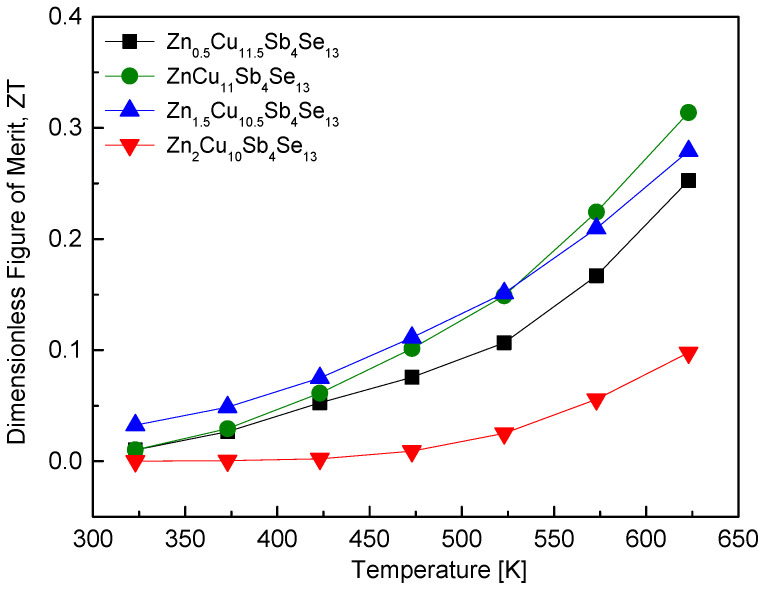
Dimensionless figure of merit for Zn_x_Cu_12−x_Sb_4_Se_13_.

Figure 11 shows the ZT values of Zn_x_Cu_12−x_Sb_4_Se_13_. With increasing temperature, the ZT increased due to the maintenance of low thermal conductivity and the rise in PF. As mentioned above, the thermoelectric characteristics of Cu_12_Sb_4_Se_13_ without the hakite phase (with permingeatite and bytizite phases only) were not measured. However, as the Zn content increased, the charge compensation partially occurred, leading to transformation into the hakite phase and the formation of permingeatite–hakite composites. In the case of ZnCu_11_Sb_4_Se_13_, high electrical conductivity and improved PF, along with low thermal conductivity, resulted in a maximum ZT of 0.31 at 623 K. However, for Zn_2_Cu_10_Sb_4_Se_13_ with complete charge compensation, the pure Zn-hakite phase was synthesized, but the ZT decreased to 0.09 at 623 K. This indicates that while charge compensation through the substitution of divalent ions is necessary for hakite synthesis, optimization of charge compensation or doping level is also required. O et al. [24] reported that FeCu_11_Sb_4_Se_13_ achieved a maximum ZT of 0.14 at 623 K in Fe_x_Cu_12−x_Sb_4_Se_13_. However, they found that as the Fe content increased, the thermal conductivity increased, leading to a decrease in the ZT value.

## 4. Conclusions

Zn-compensated Zn_x_Cu_12−x_Sb_4_Se_13_ (0 ≤ x ≤ 2) were fabricated via solid-state processing. Pure hakite (Cu_12_Sb_4_Se_13_) was unattainable; however, Zn-hakite could be synthesized where Cu was substituted (charge-compensated) with Zn. While Cu_12_Sb_4_Se_13_ contained only permingeatite and bytizite, an increase in Zn content led to the transformation of the matrix phase into hakite. All samples exhibited p-type nondegenerate semiconductor behavior. Changes in Zn content resulted in variations in electrical conductivity and the Seebeck coefficient, enhancing the power factor. Very low thermal conductivity ranging from 0.41–0.52 Wm^−1^K^−1^ was achieved at 623 K. Zn_1.5_Cu_10.5_Sb_4_Se_13_ demonstrated a maximum power factor (PF = 0.24 mWm^−1^K^−2^) at 623 K, while ZnCu_11_Sb_4_Se_13_ exhibited the highest dimensionless figure of merit (ZT = 0.31). The formation of the hakite phase was achievable through Zn charge compensation, thus providing experimental thermoelectric data based on varying Zn content. Improvement in the thermoelectric performance of synthetic hakite due to the introduction of divalent or multivalent ions was expected for further studies.

## Data Availability

The original contributions presented in the study are included in the article, further inquiries can be directed to the corresponding author.
